# Cues of Maternal Condition Influence Offspring Selfishness

**DOI:** 10.1371/journal.pone.0087214

**Published:** 2014-01-30

**Authors:** Janine W. Y. Wong, Christophe Lucas, Mathias Kölliker

**Affiliations:** 1 Department of Environmental Sciences, Zoology and Evolution, University of Basel, Basel, Switzerland; 2 Institut de Recherche sur la Biologie de l’Insecte (UMR 7261), CNRS, University of Tours, Tours, France; University of Arkansas, United States of America

## Abstract

The evolution of parent-offspring communication was mostly studied from the perspective of parents responding to begging signals conveying information about offspring condition. Parents should respond to begging because of the differential fitness returns obtained from their investment in offspring that differ in condition. For analogous reasons, offspring should adjust their behavior to cues/signals of parental condition: parents that differ in condition pay differential costs of care and, hence, should provide different amounts of food. In this study, we experimentally tested in the European earwig (*Forficula auricularia*) if cues of maternal condition affect offspring behavior in terms of sibling cannibalism. We experimentally manipulated female condition by providing them with different amounts of food, kept nymph condition constant, allowed for nymph exposure to chemical maternal cues over extended time, quantified nymph survival (deaths being due to cannibalism) and extracted and analyzed the females’ cuticular hydrocarbons (CHC). Nymph survival was significantly affected by chemical cues of maternal condition, and this effect depended on the timing of breeding. Cues of poor maternal condition enhanced nymph survival in early broods, but reduced nymph survival in late broods, and vice versa for cues of good condition. Furthermore, female condition affected the quantitative composition of their CHC profile which in turn predicted nymph survival patterns. Thus, earwig offspring are sensitive to chemical cues of maternal condition and nymphs from early and late broods show opposite reactions to the same chemical cues. Together with former evidence on maternal sensitivities to condition-dependent nymph chemical cues, our study shows context-dependent reciprocal information exchange about condition between earwig mothers and their offspring, potentially mediated by cuticular hydrocarbons.

## Introduction

Parental care evolved due to its fitness benefits to offspring, and it often comes at a cost for parents [1], [2]. Offspring that vary in condition are expected to differ in the fitness gain per unit of provisioning obtained from their parents [3], [4], [5], [6], [7], and parents that differ in condition should experience differential costs of provisioning [8], [9]. Because parents and offspring are closely related, there is an evolutionary (kin selected) incentive for parents to adjust their provisioning to offspring condition (i.e., need or quality [5], [6], [10], [11], [12]) in order to maximize their returns on investment. However, it should also pay off to offspring to adjust their demand to parental condition to moderate the cost of investment that offspring impose on their parents [8]. As a consequence, it is in the overall interest of both parents and offspring to be sensitive to variation in each other’s condition, and selection may favor the exchange of information about condition between parents and offspring through cues or signals (see [13] for definitions of terms). The evolutionary conflict between parents and offspring over parental investment [5], [10], [12], [14] may have a modulating effect in the evolution of the signals, leading to “information warfare” [15] between parents and offspring and the evolution of exaggerated and costly signals.

Previous research has focused on offspring begging signals conveying information about offspring condition as signals of need or quality [3], [6], [10], [16], [17]. The reverse expectation that offspring should be sensitive to cues of parental condition [8], or that parents even may have evolved signals to convey honest information about their condition to their offspring, has received less theoretical or empirical scrutiny. We may ultimately often expect a reciprocal form of parent-offspring communication where parents and offspring exchange information about their respective condition (and maybe even beyond, an information exchange among all family members in a communication network; [18]; see also [19], [20]). Based on these arguments, one may expect offspring to adjust their demand or selfishness to cues or signals of parental condition. The question how selfish offspring should be, how much resources they should demand from their parents, and how competitive they should be against their siblings is at the heart of parent-offspring conflict theory [5], [7], [10], [12], [14]. In its most extreme form, offspring selfishness leads to siblicide, that is, the killing and possible consumption of a sibling offspring [12], [21], [22], [23]. So, if parents provide cues or signals about their condition to their offspring (either as inadvertently released information or as evolved signal of parental condition), and offspring are sensitive to these cues, how should offspring respond in terms of their selfishness? The prediction partly depends on the consequences of the parent’s condition on the amount of obtained care, and on whether sibling interactions are purely competitive or if there is scope for cooperation among siblings (see [24] for review of evidence of sibling cooperation). Under pure competition over limited resources, offspring perceiving that their parents are in poor condition, which therefore will provide low levels of care, should compete more intensely and maybe even attempt to kill their siblings earlier (or, alternatively, disperse). This is because the poor condition of the parents would indicate insufficient resources for all offspring, enhanced sibling competition and threat of mortality. In contrast, when cooperation between offspring can compensate partly for reduced care provided by parents in poor condition, offspring perceiving cues of poor parental condition may reduce their competitive drive due to the advantage of maintaining a larger number of siblings to cooperate with. Sibling cooperation may occur for example if larger groups/broods of young are better in predator defense, have enhanced foraging efficiency or directly cooperate for example by sharing food [25], [26], [27].

Parental condition is often related to the timing of breeding, for example because individuals in good condition are able to breed early [28]. Furthermore, early breeders may face quite different ecological conditions compared to late breeders in terms of population density, food availability, predation pressure, temperature, etc., which are all factors that may also contribute to variation in their condition, in the benefits/costs of parental care (e.g., [29], [30]) and in the pay-off of sibling competition versus sibling cooperation. Correspondingly, parental cues/signals of condition and/or offspring sensitivities to these cues/signals may be expected to vary with the timing of breeding. Few studies investigated such context-dependent parent-offspring communication, but there is some evidence for different responses of parents to variation in offspring signals of quality by early and later breeders [31], [32].

The European earwig (*Forficula auricularia*) is an insect species with uniparental maternal care including egg- and offspring attendance and food provisioning [33], [34], [35], [36], [37], [38]. The offspring (nymphs) signal their condition by solicitation pheromones in the form of cuticular hydrocarbons (CHC) to which the females show two distinct responses: When exposed to CHC extracts from well-fed nymphs (as compared to poorly fed nymphs, or controls) females increase their food provisioning [39] and modify the timing of second clutch production [31]. This latter response depends on the timing of breeding, with early females advancing and late females delaying second clutch production. Furthermore, females in poor condition provide food to fewer nymphs [40] and they negatively affect their nymphs’ survival under conditions of limited food availability, probably because of mother-offspring competition over the scarce food [41]. This is in contrast to the beneficial effects of maternal presence under conditions of plentiful food where female food provisioning enhances nymph survival [34]. Finally, siblicide and cannibalism are a primary cause of mortality throughout nymph development [42], [43], which makes *F. auricularia* an ideal model system to test the influence of maternal condition cues on offspring selfishness.

## Materials and Methods

The animals used in this experiment originated from a laboratory population held according to our standard laboratory rearing protocol and based on a large founder population [44], [45]. In brief, groups of approximately 80 males and 80 females (randomly selected from the breeding stocks) were set up for mating in two plastic containers (37×22×25 cm) lined with Fluon to prevent the insects from escaping, humid sand as a substrate, and egg-cardboard and plastic tubes as shelters. The food consisted of an artificial diet [45] and was changed twice a week. The containers were kept in a climatic chamber at 60% humidity and 14 h/10 h 20°C/20°C light/dark photoperiod cycle (“summer conditions”). Upon observation of the first oviposition on 21 January 2011, all females were set up individually in Petri-dishes (10×2 cm) with humid sand as substrate and plastic shelters as nests and ad libitum food. All females were then transferred to “winter conditions”, which consisted of one week at 10°C to trigger egg-production, and 15°C afterwards and 80% humidity (throughout without light). The females were held under these conditions until the eggs hatched ( = day 0). Food was changed twice a week from isolation to oviposition. No food was provided from oviposition to hatching [34]. One day after hatching the number of hatched nymphs was counted, and the clutches were standardized to a maximum of 25 nymphs in preparation for the experimental set up (see below). The female and five randomly selected nymphs were weighed to the nearest 0.001 mg using a Mettler-Toledo MT5 Micro-balance (Mettler, Roche, Basel), provided with ad libitum food and transferred to summer conditions (see above).

### Experimental Design

The aim of the experimental design was to allow the earwig mother to release chemical cues in the substrate and to expose the nymphs to these cues over an extended time period, but preventing physical contact between mother and nymphs. We achieved this by keeping mothers and nymphs in separate Petri dishes and swapping them daily between the two Petri dishes. This treatment ensured that nymphs were exposed continuously to any chemical cues females released and left in the substrate, and that the maternal cues were renewed every other day.

The experiment was initiated on day 2 after hatching. The female and 20 nymphs (between 15 and 19 nymphs when brood size was smaller; 7 out of 37 cases) were separated and transferred to a pair of Petri-dishes (10×2 cm) containing humid sand as substrate and plastic shelters, respectively. At this stage, the females were randomly assigned either to the high food (HF) or to the low food (LF) treatment. To obtain females in HF or in LF condition, while keeping nymph condition constant, we manipulated the degree of female food access (pollen pellets [36]) and kept it constant for nymphs. HF females had daily access to large amounts of food (approx. 10 mg) for 3 hours. LF females had access only every second day to a smaller amount of food (<1 mg) for a period of 3 h (see also [40]). The nymphs had daily access to ad libitum food (pollen pellets) during these 3 h of female treatment. In all samples, the remaining food was removed after the 3 h feeding period.

Because HF females had access to larger amounts of food for a longer total amount of time, we expected them to produce more frass, which would have biased nymph food intake through allo-coprophagy and, hence, potentially nymph condition. To prevent such an effect, female frass was removed daily before swapping females and nymphs between Petri-dishes. The number of nymphs alive was counted daily. In this species, deaths due to siblicide and cannibalism cannot easily be directly observed because the attacked nymphs are consumed quickly and completely. The number of nymphs alive is therefore mostly a consequence of nymph cannibalism (only 33 dead bodies were observed over the course of the experiments; out of 721 nymphs set up in total). On day 40 after hatching, we counted the number of surviving nymphs, and we took again the weight of the female and of five randomly chosen nymphs (or fewer, depending on the number of survivors).

The sample size consisted of 37 replicates (Petri dish pairs), 18 females and their broods in the HF treatment and 19 females and their broods in the LF treatment. The experimental treatments were properly randomized as there were no significant differences between treatments in female egg-laying date (means ± s.e.; HF: 17.500±3.607, LF: 19.684±3.511; t_35_ = 0.434, p = 0.667), clutch size (HF: 67.556±2.501, LF: 63.526±2.434; t_35_ = −1.155, p = 0.256), hatching success (HF: 0.826±0.042, LF: 0.810±0.041; t_35_ = −0.279, p = 0.782), female body weight at hatching (HF: 52.178±1.805, LF: 49.826±1.757; t_35_ = −0.933, p = 0.357), or nymph body weight at hatching (HF: 1.585±0.076, LF: 1.580±0.074; t_35_ = −0.045, p = 0.965).

### Extraction and Quantification of Cuticular Hydrocarbons (CHC)

After termination of the experiment on day 40, all females were individually frozen at −30°C for later CHC extraction. For extraction, each female was immersed for 10 minutes in 800 µl of the extraction solution which consisted of n-Heptane (Rotisolv 99% pure, Carl Roth AG, Arlesheim, Switzerland) and 2.5 ng/µl n-Octadecane as an internal standard (C_18_H_38_; Fluka Analytical, Sigma-Aldrich, Buchs, Switzerland). The female was then removed from the vial and the extract stored at −30°C. Chemical analysis was carried out using Gas-Chromatography/Mass-Spectrometry (Agilent GC 7890A/5975C MSD; electron impact: 70 eV). For analysis, 2 µl extract were injected in the GC (containing 2×2.5 ng = 5 ng of the internal standard) in splitless mode (splitless time = 2 min.) and a constant inlet temperature of 250°C. The GC-MS system was equipped with a HP-5MS fused silica capillary column (length: 30 m, inner diameter: 0.250 mm, film thickness: 0.25 µm; Agilent J & W GC columns, Agilent Technologies, USA). The GC temperature program started with a temperature of 70°C (held for 2 min), then increased at 15°C/min to 232°C (held for 11 min), and then at 5°C/min to 300°C (held for 7 min). The column helium flow rate was 1 ml/min, ion detection started after a five minute solvent delay, and the MSD was set to a scan range of 40–550 m/z. For quantification of the CHC profiles, we integrated 31 peaks (of which one was the internal standard octadecane; nC18) from the chemical chromatogram using Chemstation software (Agilent Technologies, Inc.). For quantification, we divided the area of each peak by the area of the internal standard in the same chromatogram and multiplied this ratio by 5 ng to obtain an estimate of the quantity for each peak in ng. We provide peak identifications based on comparison with previous unpublished CHC identification from earwigs (Wong et al. submitted) and using fragmentation analysis [46], [47], [48] with MassHunter B.06.00 software (Agilent Technologies, Inc.). Kovats retention indices were calculated according to [49] based on a series of n-alkane standards (C8–C40, Fluka Analytical, Sigma-Aldrich, Buchs, Switzerland).

### Statistical Analysis

We analyzed the effect of the female condition treatment on the proportion of nymphs alive using a generalized linear model with a logit link, a binomial error distribution (correcting for overdispersion), the number of nymphs alive as the dependent variable, the number of nymphs originally present at experimental set up as denominator, and the female condition treatment, hatching date and their interaction as fixed effects.

The measures of peak quantities were transformed using the power transformation *y* = *x* ^0.2^ which yielded approximately normal distributions. The values *y* of each peak were then standardized to a mean = 0 and standard deviation = 1 (*z_i_* = (*y_i_-y^-^*)/*σ_y_*) [as recommended in 46]. Given the large number of peaks in the CHC data (k = 30 peaks) relative to sample size (n = 37), and in order to take into account tight correlations among individual peak quantities, we used a variable clustering approach as implemented in JMP®Pro 10.0.1 to reduce data dimensionality (for more information about variable clustering, see e.g. [50] or the SAS/STAT User’s Guide, SAS Institute Inc., Cary, NC). Variable clustering is analogous to principle component analysis, but joins highly correlated variables (pointing in a similar direction in multivariate space) in clusters [47], facilitating biological interpretation of the experimental results. After forming the clusters, the peak of each cluster that showed the strongest correlation with its own cluster as compared to the next closest cluster was used as the cluster representatives for further analysis [46].

To analyze the effects of the female condition treatment and hatching date on the female’s CHC profile we used a MANOVA with the cluster representatives as dependent variables (repeated measurements), and the treatment, hatching date and their interaction as fixed factors. To directly test for a quantitative relationship between the proportion of nymphs alive and maternal CHC we used a step-wise linear regression approach with hatching date dependent survival (see results for details on how this variable was calculated) as dependent variable and the cluster representatives as candidate explanatory variables. The model with the lowest value for the Bayesian Information Criterion (BIC) was chosen as the final model and confirmed using both forward and backward variable selection procedures. All statistical analyses were carried out using JMP®Pro 10.0.1 statistical software (SAS Institute Inc.) and all reported p-values are two-tailed.

## Results

As intended, females from the HF treatment gained significantly more weight over the course of the experiment (mean ± s.e.; 12.879 mg ±1.254) than females from the LF treatment (3.945 mg ±1.225; t_35_ = −5.088, p<0.0001), but the female food treatment did not affect nymph weight gain (from day 1 to day 40) (HF: mean ± s.e.; 3.993 mg ±0.331; LF: 4.258 mg ±0.322; t_35_ = 0.575, p = 0.569). Thus, our food manipulation successfully generated variation in female condition while keeping nymph condition unaffected.

**Figure 1:**
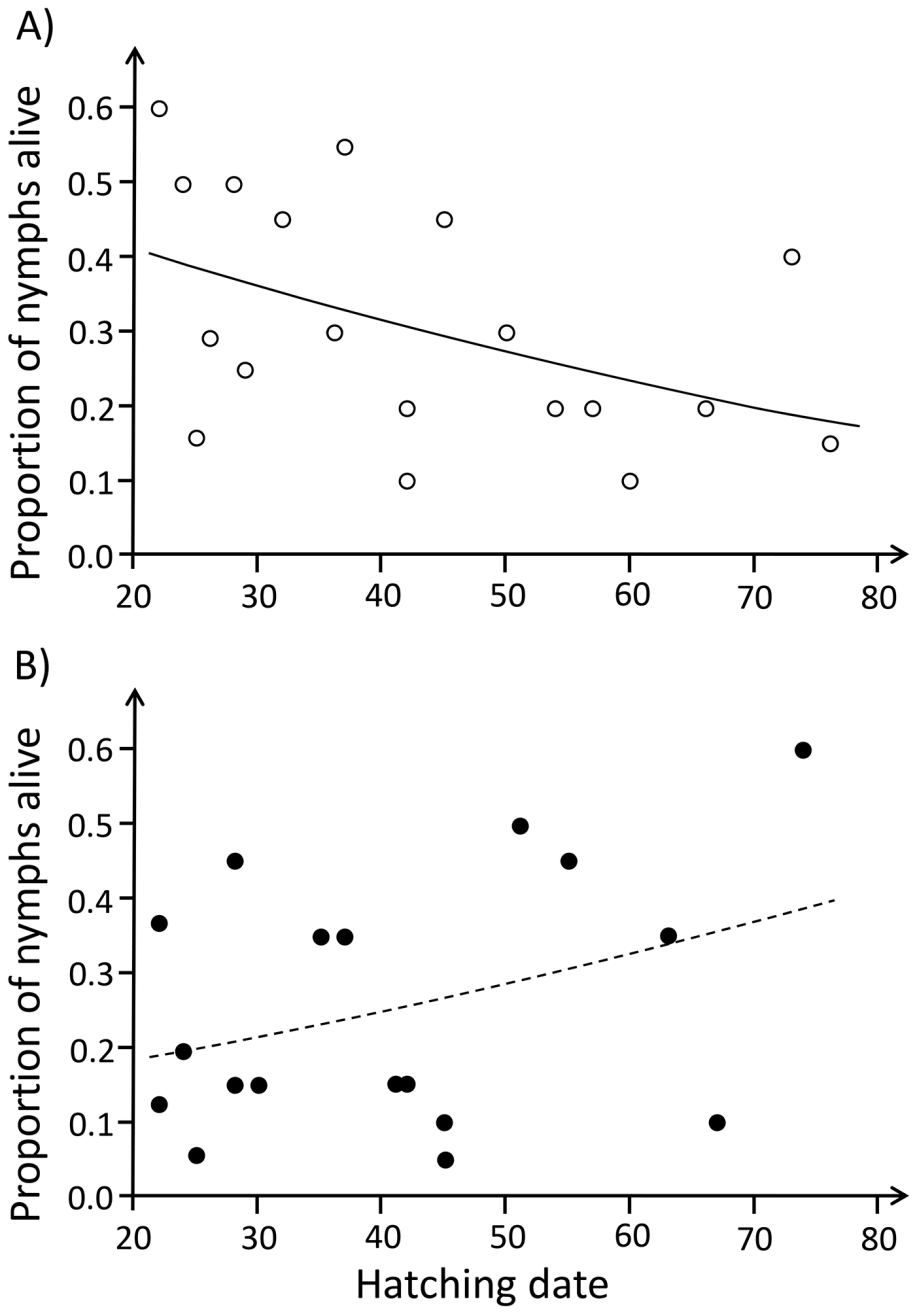
Relationship between the proportion of nymphs alive and brood hatching date for the two female condition treatments. A) low-condition treatment, B) high-condition treatment. Female condition was manipulated by varying experimentally the quantity of food to which the females had access (see Materials and Methods). On the x-axis, a julian date is provided with 6.2.2011 corresponding to day 1.

The proportion of nymphs alive on day 40 was affected by the female condition treatment through an interaction with hatching date (GLM; LR-χ^2^_1_ = 6.177, p = 0.013; Figure 1), while the main effects of the female condition treatment (LR-χ^2^_1_ = 0.899, p = 0.343) and hatching date (LR-χ^2^_1_ = 0.014, p = 0.907) were not significant. The interaction was due to a significantly higher proportion of nymphs alive in the LF treatment among early hatching broods (contrast; LR-χ^2^_1_ = 7.016, p = 0.008) and the opposite, marginally non-significant, trend among late hatching broods (contrast; LR-χ^2^_1_ = 3.456, p = 0.063) (see Figure 1).

The statistical clustering of the 30 peaks resulted in six clusters of highly correlated peaks (summarized in Table 1) jointly explaining 80.2% of the total variance in compound quantities. Entering the representative chemical compounds for each cluster (see Table 1) as repeated measures in a MANOVA with female condition treatment, hatching date and their interaction as fixed terms revealed a significant effect of female condition (but not hatching date or their interaction) on the relative CHC quantities and, hence, the composition of the CHC profile (Table 2; within-subjects effects: compound × treatment interaction). Conversely, the total CHC quantity was not significantly affected by female treatment, but dependent on hatching date (Table 2; between-subjects effects: hatching date).

**Table 1:** Summary of peaks, chemical identity of maternal cuticular hydrocarbons and their statistical clustering.

Cluster	Ret. Time	Kovats Index^2^	*Peak-ID*: Compounds	r^2^_own cluster_/r^2^_next closest cluster_/1- r^2^ ratio	Prop. Var. explained^3^
1	13.95	2098	*CC1*: nC21	0.865/0.434/0.238	0.805
	14.40	2146	*CC2*∶5-MeC21	0.824/0.757/0.724	
	14.61	2168	***CC3*** **∶3-MeC21**	0.914/0.606/0.219	
	14.88	2198	*CC4*: nC22	0.903/0.663/0.289	
	15.79	2275	CC5: X,X′-nC23∶2+ X′′-nC23∶1	0.502/0.195/0.619	
	16.05	2298	CC6: nC23	0.824/0.594/0.433	
2	16.59	2333	*CC7*∶11-, 9-, 7-MeC23	0.939/0.698/0.201	0.884
	16.77	2345	*CC8*∶5-MeC23	0.734/0.515/0.549	
	17.11	2368	*CC9*∶3-MeC23	0.941/0.757/0.241	
	17.29	2380	*CC10*: X-nC24∶1	0.865/0.451/0.246	
	17.73	2407	***CC12*** **: unknown HC**	0.939/0.698/0.201	
**3**	17.56	2397	*CC11*: nC24	0.833/0.557/0.377	0.845
	19.52	2498	*CC14*: nC25	0.704/0.338/0.447	
	20.34	2529	**CC15∶13-, 11-, 9-MeC25**	0.932/0.574/0.160	
	20.56	2537	*CC16*∶7-MeC25	0.860/0.573/0.329	
	21.34	2567	*CC17*∶3-MeC25	0.914/0.672/0.261	
	22.26	2602	*CC18*: unknown HC	0.779/0.667/0.663	
	23.33	2635	*CC19*∶13-, 11-, 9-MeC26	0.892/0.625/0.289	
4	26.30	2734	*CC22*∶13-, 11-, 9-MeC27	0.815/0.382/0.299	0.837
	26.51	2742	*CC23*∶7-MeC27	0.789/0.569/0.491	
	27.10	2764	*CC24*∶7,15-; 7,19-; 11,15-; 11,17-; 11,19-diMeC27	0.874/0.308/0.183	
	27.29	2772	***CC25*** **∶2,17-; 2,19-; 2,21-; 2,23-diMeC27**	0.871/0.269/0.176	
5	25.35	2698	*CC21*: nC27	0.199/0.011/0.809	0.704
	28.86	2838	*CC26*∶13-, 11-, 9-, 7-MeC28	0.791/0.433/0.369	
	30.94	2938	***CC28*** **∶11-, 9-, 7-MeC29**	0.946/0.140/0.062	
	31.50	2964	*CC29*∶7,19-; 9,19-; 11,17-; 11,19-diMeC29	0.878/0.144/0.142	
**6**	19.04	2473	*CC13*: X,X′-nC25∶2+ X′′-nC25∶1	0.572/0.324/0.633	0.681
	24.60	2675	*CC20*: X,X′-nC27∶2+ X′′-nC27∶1	0.775/0.508/0.458	
	29.67	2875	***CC27*** **: X,X**′**-nC29∶2**	0.785/0.272/0.295	
	33.38	3075	*CC30*: X-nC31∶1	0.590/0.077/0.444	

The representative peak for each cluster is highlighted in bold^1^. Clusters, peaks within clusters and chemical compounds within clusters are numbered according to the order of their retention times. Clusters 3 and 6 (bold) were condition dependent and significant predictors of nymph survival patterns.

1 The compound with strongest correlation with its own cluster compared to the next closest cluster (i.e., compounds with lowest 1-r^2^ ratio) were chosen as cluster representatives.

2 Index computed according to [49], and using a series of n-alkane standards (C8–C40).

3 Variance explained by the cluster divided by the total variance among the peaks of this cluster.

**Table 2:** Effect of female nutritional condition on cuticular hydrocarbon profiles.

Between-subjects effects	F_1,33_	p
Condition treatment	0.254	0.617
Hatching date	6.568	0.015
Condition treatment × hatching date	0.131	0.720
**Within-subjects interactions**	**F_5,29_**	**p**
Compound × condition treatment	5.222	0.002
Compound × hatching date	1.643	0.180
Compound × condition treatment × hatching date	0.411	0.837

Results from MANOVA with the six compound cluster representatives (see Table 1) as dependent variables (i.e., within-subjects effect) and the female condition treatment and hatching date as between-subjects effects.

**Figure 2:**
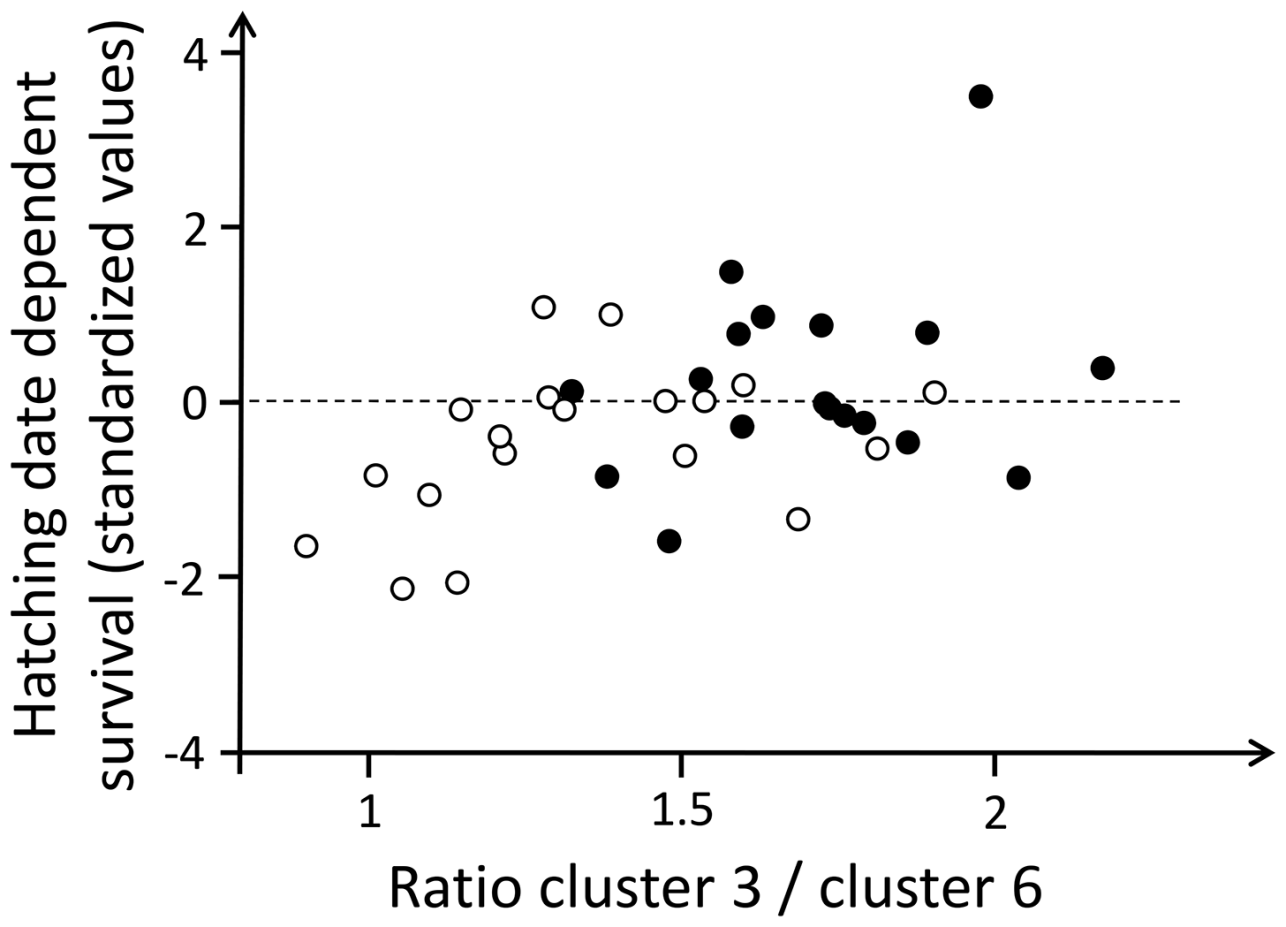
Hatching date dependent nymph survival in relation to the ratio of cluster 3/cluster 6 CHC. The hatching date dependent survival rate was computed as the product of the standardized residuals (with respect to treatment means) of the proportion of nymphs alive and of hatching date. Positive values imply lower than average survival in early hatching broods or higher than average survival in late hatching broods. Negative values imply higher than average survival in early hatching broods or lower than average survival in late hatching broods. The CHC clusters 3 and 6 were selected based on variable clustering and a step-wise linear regression (see Tables 1 & 3).

In order to correlate nymph survival patterns (Figure 1) to maternal CHC, a new variable for hatching date dependent survival was computed as the product of the standardized residuals (with respect to treatment means) of the proportion of nymphs alive and of hatching date. Positive values for this variable contribute to a positive covariance, negative values to a negative covariance between survival and hatching date. Hatching date dependent survival was significantly different between the HF and LF treatment (t_35_ = −2.151, p = 0.038; see Figure 2). To test if maternal CHC predict nymphs survival patterns, we used hatching date dependent survival as the dependent variable in a step-wise linear regression with the female condition treatment and the six compound cluster representatives as predictor variables. The final model included CHC clusters 3 and 6 (Table 1) as the sole significant linear predictor variables (positive and negative respectively; Table 3). The female condition treatment dropped from the model as its formerly significant effect was explained by these two predictors.

**Table 3:** Relationship between hatching date dependent nymph survival and female CHC.

Final model	Regressioncoefficient (± s.e.)	F_1,34_	p
CHC cluster 3	0.479 (0.165)	8.362	0.007
CHC cluster 6	−0.355 (0.165)	4.593	0.039
**Rejected terms**		**F**	
Condition treatment	–	0.332	0.568
CHC cluster 1	–	0.734	0.398
CHC cluster 2	–	0.152	0.699
CHC cluster 4	–	0.060	0.808
CHC cluster 5	–	0.598	0.445

Results from step-wise linear regression with hatching date dependent nymph survival as dependent variable (see main text for definition) and the six compound cluster representatives (see Table 1) and the female condition treatment as dependent variables. The final model (confirmed using both forward and backward model simplification) had BIC = 112.05, and r^2^ = 0.229 (null-model BIC = 114.43).

The quantity ratio of cluster 3 and cluster 6 CHC was affected by the maternal condition treatment (F_1,33_ = 11.618, p = 0.002; Fig. 2), but it was not significantly related to hatching date (F_1,33_ = 0.371, p = 0.546) or to an interaction between hatching date and treatment (F_1,33_ = 0.026, p = 0.874). Thus, the relative quantity of cluster 3 CHC compared to cluster 6 CHC was a cue for female condition and significantly predicted the hatching date dependent nymph survival pattern, but it was not in itself significantly related to hatching date.

## Discussion

Parents may transmit information about their condition or environmental conditions through pre-birth maternal effects, through their behavioral interactions with offspring, the provisioning of resources [2], [51], [52], but also through specific signals as part of a reciprocal exchange of information between parents and offspring. For example, treehopper (*Umbilia crassicornis*) nymphs signal predator threat to their tending mother through vibrational signals [53], and the mothers produce vibrational signals to reduce the likelihood of falls alarms among her nymphs [54]. In this study, we provided evidence in the European earwig *Forficula auricularia* that condition-dependent chemical cues/signals from the mother, as encoded in her CHC profile, predict offspring survival, and that the direction of this effect depended on the timing of breeding. The nymphs from both treatments had access to equal amounts of food throughout and did not differ in their weight, and cannibalism occurred in almost all cases of nymph death. Thus, the difference in survival between treatments was most likely due to variation in nymph siblicidal and cannibalistic drive, induced by cues of maternal condition.

The information transfer about female condition was not direct through a behavioral interaction from mothers to their offspring. We experimentally prevented any physical (visual, tactile, or other) contact between mothers and nymphs by keeping the mother and her nymphs in separate Petri dishes (and swapping them daily) to ensure that only chemical information about maternal condition, and not her behavior or the amount of maternal food provisioning, could mediate the observed effects on nymph siblicide and cannibalism. Thus, females must have released chemical cues in the form of non-volatile contact pheromones in the substrate, and the nymphs were exposed to these cues when subsequently placed in the same environment. Under natural conditions this indirect substrate-born signaling would occur in the breeding burrows during the period of maternal care. Female earwigs “mark” their breeding burrow with pheromone secretions (shown for the sand earwig *Labidura riparia*; [55]; pers. obs. for *F. auricularia*), to which the nymphs are then exposed while in the burrow.

The effect of maternal chemical cues of condition on nymph siblicide and cannibalism depended on the timing of breeding. Among early broods, nymphs exposed to maternal cues of poor condition showed a significantly and about two-fold higher survival rate than nymphs exposed to maternal cues of high condition. Interestingly, the effect was in the opposite direction among late broods. This effect could be either due to a quantitative or qualitative difference in the condition-dependent chemical cues among early and late breeding females or a difference in the response to the same condition-dependent cues among nymphs from early and late broods. Although our data does not allow us to fully disentangle the two possibilities, our further analyses indicate that the latter is the more likely explanation. Variation between females in CHC profiles was quantitative in nature. Early and late breeding females, and females in poor and good condition, had qualitatively the same CHC profiles. The composition of the female CHC profile (in particular the quantity ratio between cluster 3 and cluster 6 CHC) varied quantitatively with female condition, but not with timing of breeding, and it explained the treatment effect on nymph survival. We cannot fully rule out that other cues not measured by CHC extraction and GC-MS analysis (e.g., peptides or proteins) may be the causal agents underlying this effect, but any such cue would have had to be correlated with maternal CHC. Thus, our results indicate that nymphs born early and nymphs born late had opposite responses to maternal substrate-born cues of condition, expressing behavioral reaction norms [56] of opposite sign. The ratio of cluster 3 CHC quantities (mostly composed of nC25 alkanes with linear and methylated pentacosane; Table 1) to cluster 6 CHC quantities (composed of a mix of monoenes and dienes of C25, C27, C29 and C31; Table 1) was lower in females of poor condition, and was associated with lower cannibalism rates among early broods *and* higher cannibalism rates among late broods (and vice versa for higher ratios). This is evidence for context-dependence of offspring responses to maternal cues/signals. If variation in hatching date has a genetic component, these results would show genotype × family environment interactions [57] with the maternal chemical cues of condition being a component of the family environment to which the nymphs are sensitive. G × E is an important factor in the maintenance of heritable variation of phenotypic traits [58], [59] and in the present case would contribute to maintained variation in cannibalistic tendencies.

We previously showed that the same manipulation of female food access affected the food provisioning rate of earwig mothers, with females in poor condition providing food to fewer nymphs than females in high condition [40]. Furthermore, the presence of a mother can reduce nymph survival when the mother is in poor condition and food is scarce, because mothers in poor condition compete with offspring for access to the limited available resources [41]. As a consequence, nymphs should associate poor maternal condition with low expected food provisioning by their mother, and more costly interactions with her, and they should respond to the corresponding cues of maternal condition accordingly. Based on the predictions we formulated in the introduction, the higher cannibalism rate among late broods when exposed to chemical cues/signals of poor maternal condition fits a scenario of such enhanced competition when the mother is in poor condition. Conversely, the lower cannibalism rate among early broods when exposed to cues of poor maternal condition would then suggest a differential benefit of living in larger sibships and/or of sibling cooperation when the mother is in poor condition. Recent experiments demonstrated that earwig nymphs not only compete (including siblicide) [42], but that they are also very gregarious over large parts of their juvenile development [40], [60], and that they cooperate by sharing food, a behavior particularly pronounced in the absence of physical interactions with their mother [27]. Thus, there is scope for both sibling competition and cooperation in *F. auricularia*. But why should the benefits of cooperative versus competitive strategies vary with the timing of breeding? In earwigs, early broods are the first to emerge from their winter burrows and experience low densities, less cannibalism threat by other earwigs and more time for development before the next winter starts. The low density could imply that the costs of dispersing and self-foraging (to escape from a mother in poor condition with which nymphs would otherwise have to locally compete for food; [41]) may be lower for early brood nymphs. Concurrently, maintaining larger sib groups by keeping the level of siblicide low may be beneficial for self-foraging, for example because larger groups of nymphs are more efficient at foraging or provide a better protection against predators (see [61] for a review). However, further studies are required to test this hypothesis.

Our results showed that the maternal CHC profile contained reliable information about condition and was associated with time-dependent behavioral responses in offspring (i.e., cannibalistic drive) that have immediate fitness consequences in terms of survival. Thus, there is selection on this cue, and it seems likely that variation in maternal CHC profiles may have evolved to some extent due to its signaling function. We do not know if the observed variation in CHC profiles carries strategic costs (i.e., is a signal of condition) or if it rather reflects a constraint of limited food intake (i.e., is an index of condition; [13], [62]). Given that CHC derive from the fat-metabolism (which necessarily partly depends on the quality and quantity of ingested food [63]), it is possible that limitation in food intake directly constrains the quantitative production of CHC influencing CHC profiles in turn. However, the female condition treatment did not affect the overall quantity of CHC, only its composition, implying that some CHC decreased (cluster 3 CHC - nC25 alkanes with linear and methylated pentacosane; Table 1) but others increased (cluster 6 CHC - monoenes and dienes of C25, C27, C29 and C31; Table 1) under food restriction.

CHC are well known for their multitudes of functions in insect communication, especially their role as cues in insect (kin) recognition [64], [65], [66]. A comparably well studied example in the context of parental care are burying beetles (*Nicrophorus vespilloides*), where adult CHC profiles display information about breeding status (breeding versus non-breeding), and to a lesser extent also about their sex and nutritional condition [67]. Male and female parents in this biparental beetle recognize each other based on these CHC [67], [68], and CHC of adults in breeding status act as a trigger of begging behavior in the larvae [69]. However, it is not known in burying beetles if larvae modulate their begging in response to condition-dependent variation in parental CHC. CHC have been invoked as signals of quality in other social contexts. For example, in black garden ants (*Lasius niger*) it was shown that ant queen CHC convey information about queen reproductive potential, and inhibits worker ovarian development and aggression [62]. While these studies previously showed that CHC can display information about various aspects of individual condition/quality, our study suggests that CHC act as maternal condition cues mediating offspring siblicide and cannibalism and, hence, their selfishness.

## Conclusions

Taken together, our results on the effect of maternal condition-dependent cues on nymph siblicide and cannibalism reported here, and the former findings in *F. auricularia* showing that earwig nymphs express condition-dependent CHC profiles that affect maternal behavior [39] and reproductive physiology [31], we provided to our knowledge the first evidence for CHC variation to be involved in a reciprocal information exchange about nutritional condition between parents and offspring in insects. The CHC exposure effects on nymph selfishness and maternal reproductive physiology both depend on the timing of breeding. Although the ultimate causes of this variation remain to be illuminated, our findings that behavioral consequences of information exchange depend on the timing of breeding suggest that adaptive responses in communication can be strongly context-dependent and include responses that are in opposite direction.
